# Fat Fraction MRI for Longitudinal Assessment of Bone Marrow Heterogeneity in a Mouse Model of Myelofibrosis

**DOI:** 10.3390/tomography11080082

**Published:** 2025-07-28

**Authors:** Lauren Brenner, Tanner H. Robison, Timothy D. Johnson, Kristen Pettit, Moshe Talpaz, Thomas L. Chenevert, Brian D. Ross, Gary D. Luker

**Affiliations:** 1 Department of Radiology, University of Michigan Medical School, Ann Arbor, MI 48109, USA; laurendb@umich.edu (L.B.); robisont@umich.edu (T.H.R.); tlchenev@med.umich.edu (T.L.C.); bdross@umich.edu (B.D.R.); 2 Department of Biomedical Engineering, University of Michigan, Ann Arbor, MI 48109, USA; 3 Department of Biostatistics, University of Michigan School of Public Health, Ann Arbor, MI 48109, USA; tdjtdj@umich.edu; 4 Department of Internal Medicine, Division of Hematology/Oncology, University of Michigan Medical School, Ann Arbor, MI 48109, USA; krpettit@med.umich.edu (K.P.); mtalpaz@med.umich.edu (M.T.); 5 Department of Biological Chemistry, University of Michigan Medical School, Ann Arbor, MI 48109, USA; 6 Biointerfaces Institute, University of Michigan, Ann Arbor, MI 48109, USA

**Keywords:** myelofibrosis, quantitative MRI, myeloproliferative neoplasms, proton density fat fraction, hematopoietic stem and progenitor cells, bone marrow

## Abstract

Background/Objectives: Myelofibrosis (MF) is a myeloproliferative neoplasm characterized by the replacement of healthy bone marrow (BM) with malignant and fibrotic tissue. In a healthy state, bone marrow is composed of approximately 60–70% fat cells, which are replaced as disease progresses. Proton density fat fraction (PDFF), a non-invasive and quantitative MRI metric, enables analysis of BM architecture by measuring the percentage of fat versus cells in the environment. Our objective is to investigate variance in quantitative PDFF-MRI values over time as a marker of disease progression and response to treatment. Methods: We analyzed existing data from three cohorts of mice: two groups with MF that failed to respond to therapy with approved drugs for MF (ruxolitinib, fedratinib), investigational compounds (navitoclax, balixafortide), or vehicle and monitored over time by MRI; the third group consisted of healthy controls imaged at a single time point. Using in-house MATLAB programs, we performed a voxel-wise analysis of PDFF values in lower extremity bone marrow, specifically comparing the variance of each voxel within and among mice. Results: Our findings revealed a significant difference in PDFF values between healthy and diseased BM. With progressive disease non-responsive to therapy, the expansion of hematopoietic cells in BM nearly completely replaced normal fat, as determined by a markedly reduced PDFF and notable reduction in the variance in PDFF values in bone marrow over time. Conclusions: This study validated our hypothesis that the variance in PDFF in BM decreases with disease progression, indicating pathologic expansion of hematopoietic cells. We can conclude that disease progression can be tracked by a decrease in PDFF values. Analyzing variance in PDFF may improve the assessment of disease progression in pre-clinical models and ultimately patients with MF.

## 1. Introduction

Myelofibrosis (MF) is a rare myeloproliferative neoplasm (MPN) in which mutations in hematopoietic stem and progenitor cells drive unregulated expansion of myeloid lineage leukocytes, red blood cells, and platelets [1]. Diseased bone marrow (BM) in MF shows the replacement of normal fat. Fat cells typically comprise ~60–70% of bone marrow in the older adult population (median age 69 years) affected by MF, with hematopoietic cells and eventually fibrosis [2,3]. With the destruction of normal bone marrow architecture, hematopoietic stem and progenitor cells and more mature lineages disperse into other organs, such as the liver and spleen [4]. The dispersion of hematopoietic cells to other organs, termed extramedullary hematopoiesis, leads to organ enlargement, with spleen size currently serving as the standard measure for disease tracking [5]. MF patients have a median survival of 6–7 years, although substantial variation exists among patients [6].

MPNs arise from mutations in the thrombopoietin receptor (MPL), JAK2, or CALR (calreticulin) that constitutively activate JAK/STAT signaling in HSPCs [7,8,9]. Current first-generation therapies for MF, including ruxolitinib and fedratinib, target dysregulated JAK/STAT signaling [10,11]. These drugs alleviate constitutional symptoms, such as fever, night sweats, and fatigue, and reduce spleen volume [12]. However, approved drugs for MF only have minimal effects to restore normal bone marrow architecture and normal hematopoiesis [13]. The modest efficacy of current drugs for MF motivates ongoing efforts to develop more effective therapies. Investigational treatments for MF include navitoclax and balixafortide. Navitoclax is an inhibitor of B-cell lymphoma 2 (BCL-2), an anti-apoptotic protein that may induce apoptosis of malignant MF cells and improve fibrosis in BM in mouse models [14,15]. Balixafortide is a peptide-based antagonist of the chemokine receptor CXCR4, binding to its ligand, CXCL12, a chemokine that is markedly elevated in the BM of patients with MF [16,17]. Bone marrow transplant remains the only curative therapy for MF, but most patients with MF cannot tolerate this procedure.

Currently, BM biopsies are the only clinical test available to evaluate bone marrow in MF and other hematologic diseases. BM biopsy samples only a limited volume of bone marrow from one bone, the ilium [18]. Our long-term goal is to develop quantitative imaging methods to analyze the architecture of the BM for disease prognosis, prediction, and analysis of treatment response. Quantitative BM MRI provides a non-invasive approach to longitudinally analyze a much larger volume of BM than biopsy and assess multiple sites of hematopoietically active BM.

With the goal of developing quantitative BM MRI methods for MF, we previously reported studies using MRI to analyze disease status and response to therapy in mouse models of MF [19,20]. In this manuscript, we focus on developing an image analysis method to differentiate healthy versus diseased BM based on variance in values for one MRI parameter, proton density fat fraction (PDFF). PDFF distinguishes protons associated with fat versus water based on differences in chemical shift by MRI [21]. Since cells comprise the major source of water in BM, PDFF measures relative amounts of fat versus cells (water) in each voxel. To extract more diagnostic information from images, we performed a voxel-wise analysis of PDFF images within and among various imaging time points for mice with MF. We determined the variance in PDFF for all voxels within regions of hematopoietically active BM in mouse tibias. We then compared these values over time. Relative to the high variance in PDFF in healthy BM due to the normal heterogeneity of cells and fat, the expansion of hematopoietic cells in the BM of mice with MF showed a pronounced reduction in the variance in PDFF values. These results demonstrate an image analysis method that may improve the detection of disease status and response to therapy with bone marrow MRI for MF. 

## 2. Materials and Methods

We analyzed MRI data from mice with MF generated using a BM transplant model in which we transduced hematopoietic stem and progenitor cells (HSPCs) ex vivo with a retrovirus expressing a mutant MPL receptor (MPLW515F) known to drive MF in patients [22]. We selected the MPL driver mutation because it causes the most aggressive subtype of myeloproliferative neoplasms (MPNs). We transplanted transduced HSCs via tail vein injection into 8–10-week-old male or female BALB/c mice (Charles River Laboratories) after sub-lethal treatment with 2 × 4.5 Gy gamma irradiation separated by 24 h. We matched the gender of donor HSPCs and recipient mice. We are reanalyzing data from previous experiments by our group, as well as comparing data from new studies. We previously reported the full experimental setup for these studies [19,20].

### 2.1. Study Populations

We analyzed data from three independent study groups: 1 healthy control group and 2 groups with diseased bone marrow. Mice were classified as diseased if there was an increase in spleen size, the PRM showed a decrease in PDFF, and a decreasing trend in PDFF based on the axial mean trajectory over the study period. Disease 1 included mice with MF that acted as vehicle control or that did not respond to treatment, as assessed by the change in spleen volume and PDFF in BM. We started therapy after the mice had established disease, as documented by imaging spleen volume and marrow. After documenting the presence of MF, we randomly assigned mice to receive ruxolitinib, fedratinib, or navitoclax as described, with imaging studies on days 0 (baseline after irradiation and disease transplant), 64, 69, and 74 [20]. Disease 2 was a cohort of mice with MF that did not respond to treatment with ruxolitinib, balixafortide, both ruxolitinib and balixafortide, or the vehicle. Imaging studies of these mice occurred on days 0 (baseline after irradiation and disease transplant), 55, 77, and 93. We administered balixafortide subcutaneously daily at a dose of 20 mg/kg. We selected a randomized cohort of mice from each of these treatment groups. Disease group 1 and disease group 2 were gathered from two studies conducted independently. We used data from both studies to increase repeatability and further validate our testing methods. Both studies employed the same analysis techniques and testing procedures.

### 2.2. MRI Parameters

We quantified spleen volume, a standard marker of disease severity in MF, and PDFF in the BM of the tibia using a 7 T, 30 cm bore MRI scanner (Bruker BioSpec Paravision version 7.0.0), as described previously. For spleen imaging, we used an 86 mm diameter radiofrequency transmit/receive coil. To image the tibia, we utilized a 4-channel cryogenically cooled surface coil. We acquired PDFF images with a 3D multi-echo, gradient-echo (TR = 50 ms, TE = 1.47 + [*n* × 0.0317] ms, where *n* = 0, 1, … 11) sequence [20].

### 2.3. Image Analysis

We used in-house MATLAB scripts to segment the BM and isolate the region of interest (ROI) for the BM. This study focuses on tracking PDFF, as our previous work has shown it to be indicative of both therapeutic response and disease progression. Although various imaging modalities were collected throughout the study, we prioritized PDFF due to its consistency across images and prior publications, demonstrating its reproducibility and clinical relevance [23]. The distal tibia in healthy mice shows predominantly fat, unlike the proximal tibia, which contains predominantly hematopoietic BM. Therefore, we analyzed BM only in the distal tibia as PDFF cannot distinguish between normal and malignant hematopoietic cells in the proximal tibia. We initially manually drew the ROI to encompass the entire BM, followed by computationally segmenting the BM into the proximal, transition, and distal regions. After defining the axial MRI slice that marked the distal region, we removed the proximal and transition regions of the ROI. To ensure proper spatial alignment longitudinally, we registered the images using Elastix [24,25]. We used an in-house Transformix protocol to apply the mask to all image types acquired throughout the study.

We calculated the variance of each voxel in the ROI using a custom MATLAB (R2019b, Mathworks, Natick, MA, USA) script and extracted the 90th percentile of these values to remove extreme outliers. We exported the filtered data to Excel to calculate the variance among mice at each time point. We generated heatmaps of PDFF values in the mouse tibia over time using in-house MATLAB scripts. We directly visualized tibia BM by histology with staining for reticulin as a marker of fibrosis and hematoxylin and eosin staining as described previously [19].

### 2.4. Statistical Analysis

To determine to what extent the diseased and healthy groups of mice differed, we used a custom MATLAB script to run a permutation test and principal component analysis (PCA) at a significance value of 0.05. We compared disease 1 and disease 2 with a nonparametric two-sided test. We compared both disease groups against healthy individuals via a nonparametric one-sided test. We compared disease groups 1 and 2 by principal component analysis (PCA) in a 2D plot. We used a single time point, 1D PCA, to compare the healthy group with both disease groups combined.

Using GraphPad Prism 10 (La Jolla, CA, USA), we constructed violin plots of the variance values for each group. Each time point on the violin plot represents the spread of variances across individual mice at that time.

We used a custom linear regression script in MATLAB to determine the values for the line of best fit. We plotted the variance at each time point for each mouse and calculated the corresponding line of best fit, which we then plotted in GraphPad Prism 10.

## 3. Results

**Figure 1:**
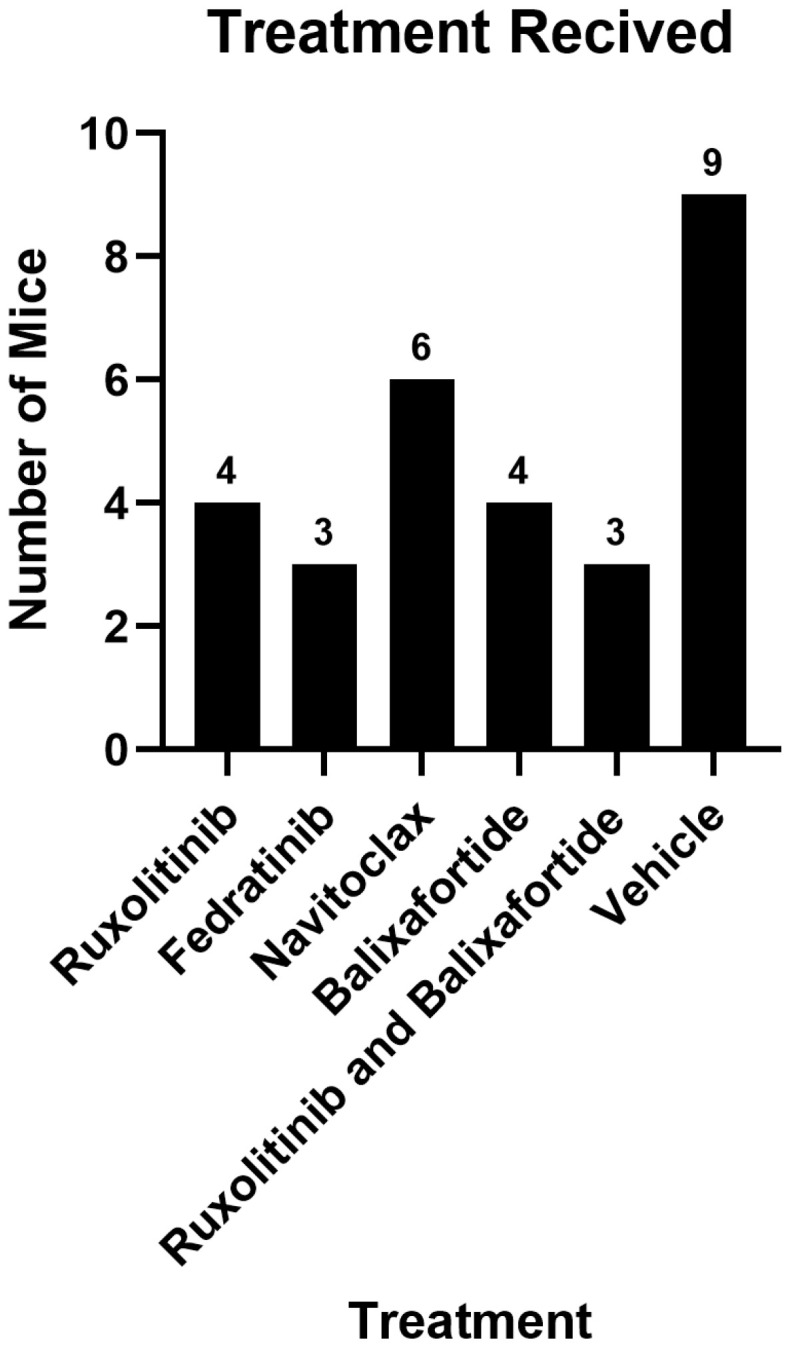
**Study population for disease groups non-responsive to administered treatments.** Disease group 1 received ruxolitinib, fedratinib, navitoclax, or vehicle control. Disease group 2 received ruxolitinib, balixafortide, both, or vehicle control. We randomly selected mice from each disease cohort, and among treatments, with the sole inclusion criterion being failure of treatment to reduce splenomegaly or hypercellular bone marrow as determined by MRI. All mice from the healthy group were at pre-treatment time points and are not represented in this figure.

We reanalyzed data from three groups of mice in this study: disease 1, disease 2, and healthy. Disease 1 and disease 2, respectively, included mice with MF from two independent cohorts that did not respond to therapy as defined by no significant change in spleen volume or a decrease in PDFF in the distal tibia. Mice in the disease cohorts either received the vehicle control or were unresponsive to the treatment administered (Figure 1). The treatments the mice received were randomized, and mice were selected independently of the therapies administered. This figure highlights the broad applicability of our methods, demonstrating that the bone marrow response is not influenced by the type of therapy. The healthy group consisted of a single time-point MRI study performed on healthy mice before irradiation and bone marrow transplant. We also show selected examples of mice with favorable responses to treatment as defined by a 50% reduction in spleen volume.

**Figure 2:**
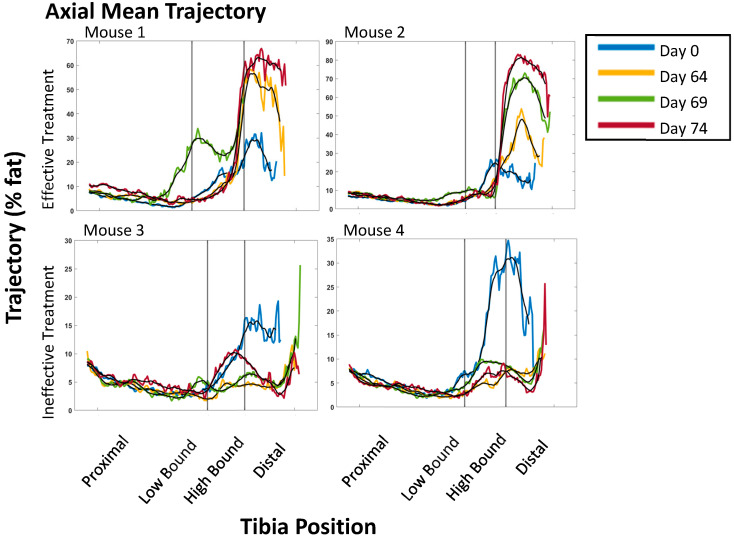
**Effective therapy increases PDFF values in the distal tibia.** Axial mean trajectory graphs for two different mice undergoing effective (**top**) and ineffective (**bottom**) treatment. The baseline time point (day 0) is in blue, day 64 in yellow, day 69 in green, and day 74 in red. The grey lines denote the BM region: left is proximal, middle is transition, and right is the distal region. Note that the slight differences in the *y*-axis are due to the biological variability in the distal region of each mouse. The proximal region on the left of the graph shows minimal change due to the proximal tibia containing normal hematopoietic cells indistinguishable from malignant cells, as determined by PDFF-MRI. We did not analyze the middle transition region due to the high noise. The distal region on the right side of the graphs demonstrates an increase in PDFF values for effective treatment and a decrease in PDFF values for ineffective treatment. We focused this study on the distal region because the expansion of malignant hematopoietic cells in MF replaces normal BM fat as the disease progresses.

We used axial mean trajectory plots of PDFF values to define proximal, transition, and distal regions of the tibia at each time point in the study [19]. Unlike adult humans, the proximal tibia in mice contains hematopoietically active BM, meaning that the percentage of fat is very low (Figure 2) [26]. The distal tibia BM predominantly contains fat in healthy mice, making this a site where MRI can detect the loss of BM fat with MF and the re-emergence of fat with effective therapy. We did not analyze the transitional middle region of the BM due to high variability in PDFF values [19].

### 3.1. PDFF Image Analysis for Heterogeneity

**Figure 3:**
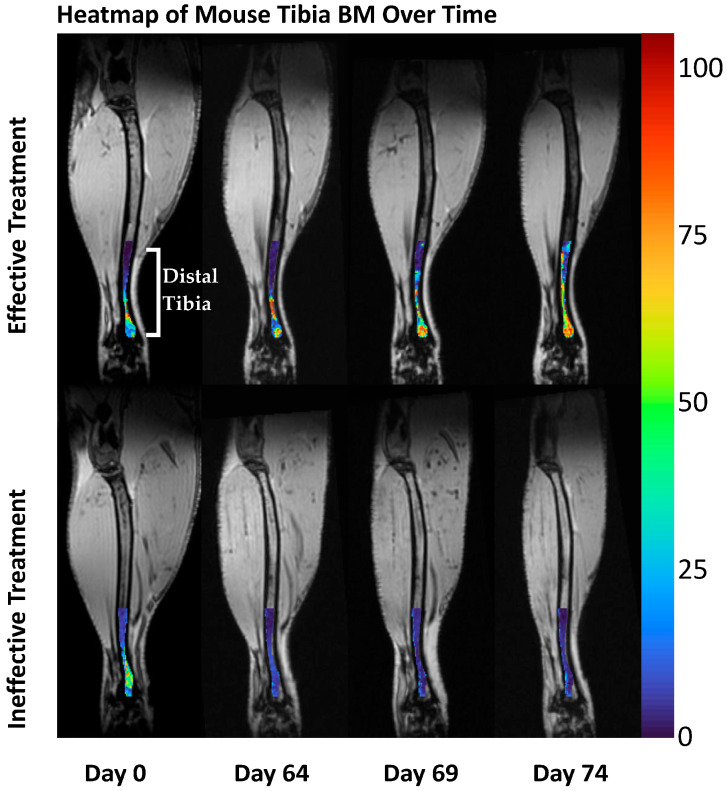
**Ineffective treatment results in low variance in PDFF values in the distal tibia of mice with MF.** Heatmap of the mouse tibia over the study period of 74 days. Values of 0 and 100 define no and 100% fat cells, respectively. Effective treatment (**top**) shows an increasing number of fat cells as the BM progresses towards a healthy baseline. Ineffective treatment (**bottom**) shows almost no heterogeneity with low amounts of fat as measured by PDFF. Muscle tissue may appear different for each scan date due to the positioning of the tibia in the coil, but the same slice is displayed for each time point. The PDFF ROI and heatmap are overlayed onto the T1-weighted anatomic image for display clarity.

To further visualize the composition of and variability in PDFF in BM, we constructed heatmaps displaying the range of values on a pseudo-color scale. Healthy mice (day 0) showed heterogeneous PDFF values at baseline. Heterogeneity in PDFF in the distal tibia remained evident in mice with effective treatment, independent of the administered compound. Diseased mice exhibited high variability at day 0 in the distal tibia with decreasing PDFF values approaching zero over time. In an effectively treated mouse, as defined by a 50% reduction in spleen volume, PDFF values increased over time as the number of fat cells and variance in the BM increased back towards a healthy baseline (Figure 3).

**Figure 4:**
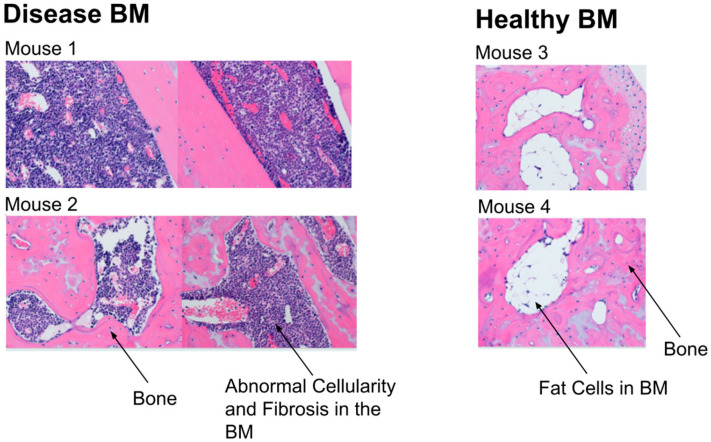
**Histology shows the replacement of normal bone marrow fat in the distal tibia BM in mice receiving ineffective treatment for MF.** Pink areas represent collagen in bone; white shows fat cells in healthy mice; and purple areas show hypercellular bone marrow. Mice with MF show hypercellular bone marrow, replacing the normal fat cells and other stromal cells seen in healthy mice.

By histology with hematoxylin and eosin staining, we observed hypercellular BM with complete replacement of fat in mice with MF (Figure 4). Healthy mice showed the opposite finding, with large amounts of BM fat. These histology images validate MRI data for low and high PDFF values in the distal tibias of mice with MF versus healthy controls, respectively.

### 3.2. Disease Groups Show Comparable Imaging Findings

To conduct a voxel-wise comparison between treatment groups, we first calculated the variance of each voxel within the ROI for each PDFF image. We then took the 90th percentile of these values to remove any extreme outliers. Then, for each time point in each mouse, we calculated the variance of these voxel-wise variances within the ROI. The healthy group was examined during a single-time-point study. Table 1 shows the raw variance values for each treatment group.

**Figure 5:**
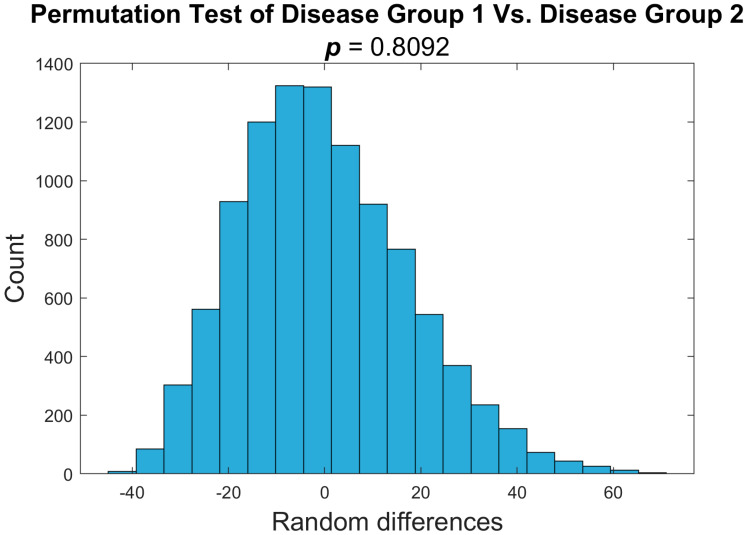
**Disease 1 and disease 2 cohorts of mice show no difference in PDFF variance.** Nonparametric two-sided permutation test to determine significant difference between disease 1 and disease 2 groups. *p*-value of 0.8092 at a significance level of 0.05. There is no significant difference between the two diseased cohorts of mice, allowing us to combine these groups into one disease cohort for analysis.

**Figure 6:**
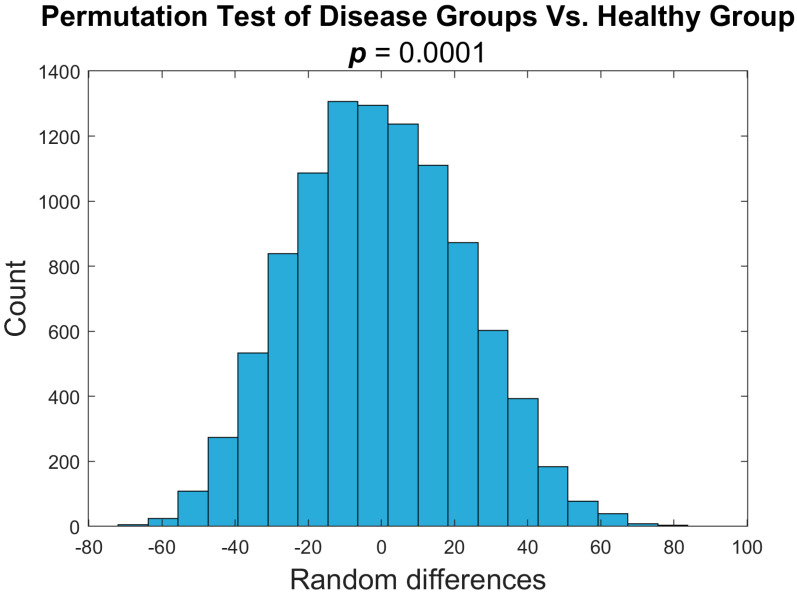
**Mice with MF significantly differ from healthy mice by variance in PDFF MRI.** Nonparametric one-sided permutation test between pre-treatment MF disease and healthy group baseline values. Variance in PDFF values differs significantly between groups (*p* = 0.0001).

We used a nonparametric permutation test to determine any significant differences in PDFF values between the disease 1 and disease 2 groups. We determined that the two groups did not differ significantly (*p*-value < 0.05 (Figure 5)). The *p*-value of 0.81 did not allow us to reject the null hypothesis, proving there was no distinguishable difference between the two groups. This analysis allows us to combine the two disease cohorts in later analyses. To test the difference between the disease groups and healthy mice, we combined pre-treatment time points for both MF disease groups and compared these values against the healthy group in a one-sided nonparametric permutation test. We determined that the two groups did differ significantly (*p*-value < 0.05 (Figure 6)). This data demonstrates a distinguishable difference between the healthy and disease groups. This analysis shows that a quantitative voxel-wise comparison of the heterogeneity through MRI-PDFF in the bone marrow can be indicative of disease status.

**Figure 7:**
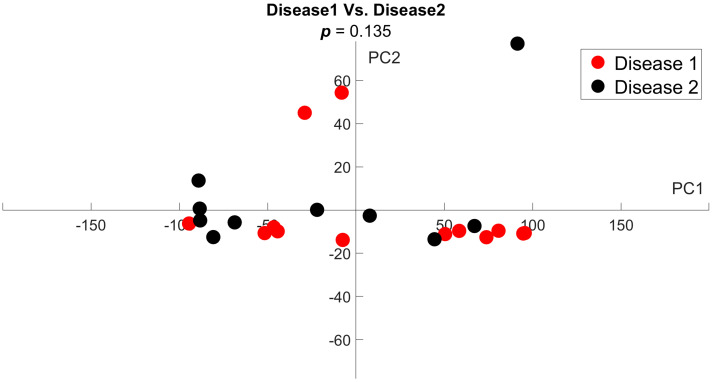
**Principal component analysis reveals no difference in variance in PDFF MRI values for disease 1 and 2 groups.** We performed PCA to assess variance patterns across time points for each mouse within the disease 1 and disease 2 cohorts. The lack of clustering or distinction between the two treatment groups shows that the disease groups can be grouped into one cohort for further analysis (*p* = 0.135).

**Figure 8:**
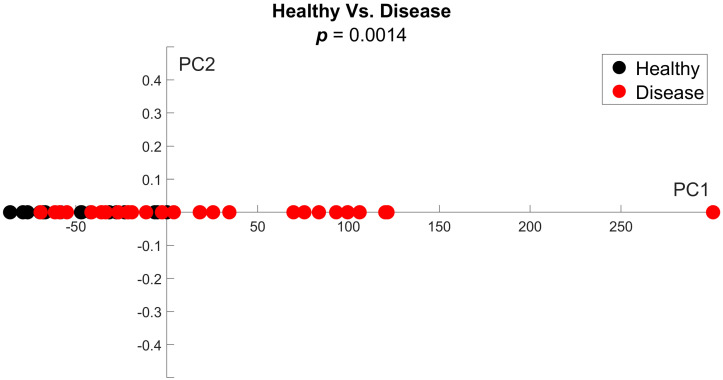
**One-dimensional principal component analysis shows a large difference in PDFF variance between the combined disease and healthy groups.** Variance in PDFF values for the combined disease groups spans the entire *x*-axis. The healthy values cluster on the negative *x*-axis, exhibiting a significant difference (*p* = 0.0014).

We further compared the combined MF disease groups and the healthy group by principal component analysis (PCA). When comparing disease 1 versus disease 2, we did not see significant clusters of either group, showing that these groups are indistinguishable from each other (*p*-value < 0.05 (Figure 7)). Significant outliers in both groups along PC2 show the biological variability within diseased BM. A one-dimensional, single-time-point PCA comparing the combined disease groups and the healthy group revealed that all healthy data clustered closely on the negative *x*-axis. The disease data points show greater divergence, covering a large portion of the positive *x*-axis. These data further reinforce our conclusion that PDFF MRI shows a significant difference between the disease groups and the healthy group (*p*-value < 0.05 (Figure 8)).

The results of the permutation tests and PCA demonstrate the repeatability and reproducibility of the findings in this study. Given the lack of a distinguishable difference between the two separate disease cohorts, the trends observed in disease BM appear consistent across studies performed at different times. These data support the validity of combining disease groups in future analyses, enabling more significant comparisons against the healthy group.

### 3.3. Variance as an Indicator of Disease Status

**Figure 9:**
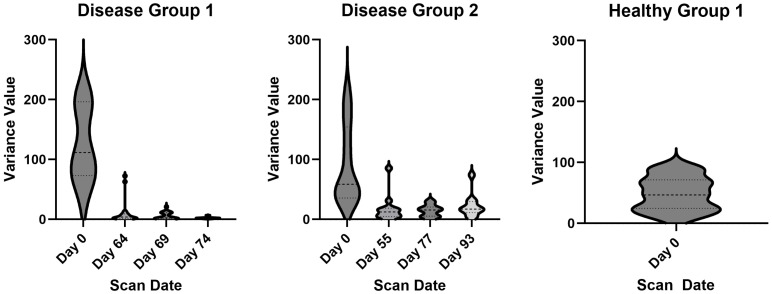
**Hypercellular BM in mice with MF shows minimal variance in PDFF values.** For each mouse, we calculated the variance of every voxel at each time point, presented as violin plots to show variability of these values. The *y*-axis is capped at 300 to maintain a consistent scale across all groups; however, note that some outliers in disease groups 1 and 2 exceed this limit. The dashed lines represent the interquartile range. The bottom line is Q1, the middle line is median and the top line is Q3. Thelarge spread in day 0 in the disease groups is due to the irradiated BM, with a drastic drop in variance as disease state progresses. The healthy group shows a spread in variance from 0 to 100 (mean 49.0%) fat cells in the BM.

To further analyze the variance within groups, we constructed violin plots. In the disease groups, the plots show a large spread in PDFF values initially in mice (day 0), followed by a marked decline in variance with progression of MF (Figure 9). The large range in variance on day 0 is due to irradiated BM for the transplant procedure. At this time point, the BM has no composition and therefore lots of variability. By the second scan date in both cohorts, mice developed MF with hypercellular bone marrow replacing fat. A total of 84.8% of the variance values for PDFF after the baseline time point are below 10%. In the healthy group, all the PDFF values are in the range of 0 to 100 with an average value of 49.0% of the BM as fat cells (Figure 9).

**Figure 10:**
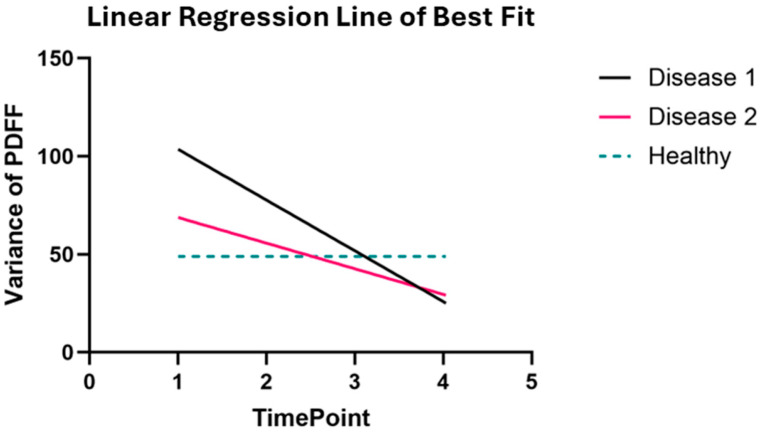
**Variance in PDFF decreases in mice that are non-responsive to treatment for MF.** We generated lines of best fit by combining the linear regressions of each mouse’s variance over time. The healthy single-time-point study shows an average variance value across all samples of 49.0%. The large variability in the disease groups can be seen by the high initial value and rapid negative slope. The disease BM surpasses the healthy baseline as fat cells are replaced by hypercellular bone marrow with unregulated proliferation of hematopoietic cells.

To demonstrate the relationships within each disease group, we performed a linear regression analysis to capture the line of best fit for each group. The regression analysis depicts the large variability and rapid decrease in variance in the disease groups. These groups started at a high variance value and then quickly dropped to approximately zero. The average value of the healthy group is plotted across the time points, showing the healthy baseline of ~49.0% (Figure 10).

## 4. Discussion

A key feature of MF is the destruction of normal BM macroscale architecture. The dysregulated proliferation of myeloid lineage cells replaces normal BM fat cells, the predominant cell type in hematopoietically active adult bone marrow. As the disease progresses, the progressive deposition of reticulin and collagen fibrosis and osteosclerosis (thickening of the bone structure) further disrupt normal BM architecture [27]. Because of abnormalities in BM, hematopoiesis shifts to the liver and spleen, sometimes causing massive enlargement of these organs. Clinical trials and clinical oncology currently use reductions in spleen volume as a marker of treatment efficacy [28]. Not surprisingly, first-generation approved drugs for MF shrink the spleen but have minimal or no effect in restoring normal BM architecture and composition. The current emphasis in drug development for MF centers on reversing pathological changes in BM to improve outcomes for patients [29]. However, bone marrow biopsy, the current clinical approach for assessing BM in MF and other hematologic malignancies, suffers from the notable limitations of sampling error and limited patient tolerance as a tool to monitor the effects of therapy on BM. To help overcome the limitations of BM biopsy to monitor treatment, we are developing quantitative BM MRI methods as a non-invasive approach to assess the effects of therapy in pre-clinical mouse models and patients over time in MF.

To develop improved image analysis methods for BM MRI, we focused on data on PDFF in mice with MF or healthy controls. Since one of the major pathologic changes in BM in MF is the replacement of normal BM fat with hematopoietic cells, PDFF provides a non-invasive approach to quantify this key marker of disease. We assessed three groups of mice: two cohorts with MF (disease 1 and disease 2) and a healthy control group. The MF disease groups consisted of two cohorts of mice that did not respond to treatment with various approved drugs and investigational compounds for MF, which we scanned by MRI at different times. We analyzed the MRI scans of these mice with MF performed at various times in therapy, allowing us to assess changes in PDFF over time. Our analysis confirmed no difference between these cohorts of diseased mice, allowing them to be combined into a single disease group for subsequent investigation. The healthy group is a single-time-point study of mice imaged before disease induction.

The combined disease groups exhibited a large variance in PDFF at day 0 due to BM irradiation for the transplant procedure. However, as the disease progressed, the PDFF in the distal tibia decreased towards zero, indicating the replacement of normal bone marrow fat with malignant hematopoietic cells. We focused on the distal tibia, as only this part of the bone normally contains substantial fat (~50%), enabling us to detect the expansion of hematopoietic cells. Consistent with visual observations of PDFF, voxel-wise longitudinal analysis of BM PDFF values in the distal tibia showed a marked reduction in variance of these values in mice with treatment refractory MF. The variance in PDFF in mice with MF showed notably lower values than healthy mice, permitting us to clearly distinguish diseased from normal BM. We validated MRI data with histology in the current study and our prior work [19,20].

We previously used a similar voxel-wise analysis technique, termed parametric response mapping, to quantify changes in imaging parameters over time [30,31]. Parametric response mapping improves the detection of response to therapy relative to whole-tumor or region mean values. A limitation of parametric response mapping is the need to exactly co-register and match individual voxels on serial imaging studies. The need for the co-registration of each voxel increases computational time and creates challenges if the overall ROI changes in serial imaging studies, which necessitates the warping of images and voxels. In the case of our studies using skeletally immature mice, the growth of the tibia throughout the experiment changed the overall volume and dimensions of the tibia bone marrow. Similar effects could occur in patients where progressive osteosclerosis in advanced MF could alter the size of the bone marrow space in various bones.

Analyzing the variance in PDFF or other values within the ROI in sequential imaging studies represents a middle ground in image analysis. This approach still captures heterogeneity within the ROI rather than masking heterogeneity with an average value; yet it measures changes in the variance and speed of imaging by eliminating the need for the exact co-registration of voxels. Analyzing variance in BM PDFF and other BM MRI parameters may potentially improve the detection of response to therapy and restoration of BM architecture. Future studies will address the utility of this image analysis approach in our ongoing clinical MRI study of patients with MF and determine to what extent changes in variance improve treatment monitoring as compared with changes in spleen volume, the current metric for treatment efficacy.

### 4.1. Current Applications of Quantitative PDFF MRI

Quantitative PDFF-MRI is also used to track fat content in the early phase of liver disease [32,33]. Early-phase trials for nonalcoholic steatohepatitis (NASH) use MRI metrics as a non-invasive means to track disease prognosis and response to treatment [34]. PDFF reliably detected liver fat content over longitudinal imaging studies, making it a non-invasive surrogate for liver biopsy. A decrease in PDFF corresponded to higher chances of histologic response on liver biopsy and positive treatment effects [33]. Similarly, while PDFF for bone marrow likely will not replace biopsy for assessing bone marrow in MF, we envision that this quantitative MRI method may greatly reduce or potentially eliminate the need for biopsy to determine response to therapy.

### 4.2. Limitations

A limitation of this study is the modest sample size in the study cohorts. Disease 1 had a sample size of 17, disease 2 had a sample size of 12, and the healthy group had a sample size of 17. Challenges with increasing sample numbers include the labor-intensive BM transplant procedure needed to generate mouse models of MF and the approximately one-hour scan time for each MRI per mouse. To increase the size of cohorts, we grouped mice that failed to respond to any of several different treatments. Typical treatment studies in BM transplant mouse models of MF begin therapy within 2 weeks of the transplant, more closely aligning such studies with disease prevention rather than the clinical scenario of treating persons with established disease. Delaying the onset of therapy accounts for the very low response rates in the mice analyzed for this work [14]. We have also demonstrated that beginning treatment with ruxolitinib within 2 weeks of a bone marrow transplant produces nearly complete responses in MRI and histology [13]. Using a genetically engineered mouse model of MF in future studies will potentially allow us to generate larger cohorts of mice and statistically meaningful numbers of mice responding to therapy.

Lastly, other limitations included that there was imaging only the tibia of mice and the sole focus on PDFF. Because of the small size of mouse bones, we use a cryogenically cooled surface coil to enhance signal-to-noise and improve spatial resolution. Our coil fits the mouse tibia, and imaging this bone also eliminates problems with respiratory motion artifact. Our human studies include larger volumes of bone marrow from different sites, which better captures the heterogeneity of MF [17]. We aim to further evaluate human bone marrow in future studies to support the translation of this method into clinical practice. We have reported other MRI parameters for imaging BM, including apparent diffusion coefficient (ADC) and magnetization transfer ratio (MTR). We chose to focus only on PDFF due to the direct correlation between healthy BM exhibiting large numbers of fat cells and the high signal-to-noise and spatial resolution of this sequence [19]. Future studies with larger sample sizes will attempt to include and combine additional imaging parameters.

## 5. Conclusions

Overall, this study shows that analyzing the variance in PDFF values in BM distinguishes diseased from healthy BM in mouse models of MF. This data further supports the potential future application of quantitative MRI methods to assess BM in patients undergoing therapy for MF. We envision that measuring the variance in PDFF and other MRI metrics will improve the detection of response to therapy by detecting changes in variance over time for each subject. Quantitative BM MRI shows promise as a non-invasive complement to biopsy to assess the macroscale architecture of BM in clinical trials and clinical oncology for MF.

## Figures and Tables

**Table 1:** Raw variance values of the three treatment groups over the study period. The tables present the raw variance values of the PDFF in the BM for each treatment group. Each table is organized with individual mouse numbers listed in the leftmost column and scan dates displayed across the top row. The table on the left shows baseline variance values from the healthy group, scanned once prior to malignant cell transplantation. The center table contains values from disease group 1, and the rightmost table presents data from disease group. Note that disease groups 1 and 2 were part of two independent studies, resulting in slight differences in scan dates and intervals. Although mouse numbers vary across groups, all available data was included due to the small sample size overall. Empty cells indicate mice that did not survive through the entirety of the study period; the data that was collected is included.

Healthy		Disease 1					Disease 2				
Mouse	Day 0	Mouse	Day 0	Day 64	Day 69	Day 74	Mouse	Day 0	Day 55	Day 77	Day 93
Mouse 1	65.89	Mouse 1	82.12	1.05	1.26		Mouse 1	31.93	9.5	18.89	19.77
Mouse 2	14.32	Mouse 2	111.74	1.57	20.78	1.65	Mouse 2	57.44	31.09		
Mouse 3	34.97	Mouse 3	214.96	1.4	10.82	6.71	Mouse 3	163.22	1.41	1.6	3.29
Mouse 4	61.81	Mouse 4	199.62	4.41	4.83	1.96	Mouse 4	38.51	3.59	7.05	13.59
Mouse 5	25.33	Mouse 5	66.81	7.55	0.74	2.04	Mouse 5	52.02	5.98	20.52	28.64
Mouse 6	70.4	Mouse 6	177.22	5.16	1.65	2.37	Mouse 6	127.93	9.52	15.55	17.99
Mouse 7	46.29	Mouse 7	72.02	10.33	1.42	0.93	Mouse 7	97.35	16.15	14.07	4.62
Mouse 8	72.34	Mouse 8	111.65	72.55	2.75	2.83	Mouse 8	59.87	19.28	5.45	
Mouse 9	26.59	Mouse 9	213.76	2.53	9.85	0.87	Mouse 9	34.51	16.19	27.19	74.25
Mouse 10	86.88	Mouse 10	169.2	3.93	1.18	2.26	Mouse 10	186.73	4.13	3.3	16.2
Mouse 11	16.88	Mouse 11	118.98	2.35			Mouse 11	31.97	15.13	31.33	15.4
Mouse 12	23.8	Mouse 12	24.07	13.27	2.05	2.11	Mouse 12	213.79	85.38	16.48	32.83
Mouse 13	88.29	Mouse 13	74.3	7.85	0.68	3.62					
Mouse 14	46.58	Mouse 14	193.03	0.83	13.17	2.38					
Mouse 15	93.63	Mouse 15	394.17	1.86	0.59	0.62					
		Mouse 16	90.85	62.97	11.41	2.86					
		Mouse 17	57.7	0.6	12.34						

## Data Availability

We will make all imaging data and MATLAB code available upon reasonable request.

## Abbreviations

The following abbreviations are used in this manuscript:

PDFF	Proton Density Fat Fraction
MF	Myelofibrosis
BM	Bone Marrow
HSPC	Hematopoietic Stem and Progenitor Cells
MPN	Myeloproliferative Neoplasm
ROIs	Region of Interest
PCA	Principal Component Analysis
ADC	Apparent Diffusion Coefficient
MTR	Magnetization Transfer Ratio
NASH	Nonalcoholic Steatohepatitis
